# Metabolic Dysfunction Underlying Autism Spectrum Disorder and Potential Treatment Approaches

**DOI:** 10.3389/fnmol.2017.00034

**Published:** 2017-02-21

**Authors:** Ning Cheng, Jong M. Rho, Susan A. Masino

**Affiliations:** ^1^Departments of Pediatrics, University of CalgaryCalgary, AB, Canada; ^2^Clinical Neurosciences, University of CalgaryCalgary, AB, Canada; ^3^Physiology and Pharmacology, Alberta Children's Hospital Research Institute, Cumming School of Medicine, University of CalgaryCalgary, AB, Canada; ^4^Neuroscience Program, Department of Psychology, Trinity CollegeHartford, CT, USA

**Keywords:** autism spectrum disorder, ketogenic diet, metabolism, mitochondria, therapeutics, epilepsy, co-morbidity, mechanism

## Abstract

Autism spectrum disorder (ASD) is characterized by deficits in sociability and communication, and increased repetitive and/or restrictive behaviors. While the etio-pathogenesis of ASD is unknown, clinical manifestations are diverse and many possible genetic and environmental factors have been implicated. As such, it has been a great challenge to identify key neurobiological mechanisms and to develop effective treatments. Current therapies focus on co-morbid conditions (such as epileptic seizures and sleep disturbances) and there is no cure for the core symptoms. Recent studies have increasingly implicated mitochondrial dysfunction in ASD. The fact that mitochondria are an integral part of diverse cellular functions and are susceptible to many insults could explain how a wide range of factors can contribute to a consistent behavioral phenotype in ASD. Meanwhile, the high-fat, low-carbohydrate ketogenic diet (KD), used for nearly a century to treat medically intractable epilepsy, has been shown to enhance mitochondrial function through a multiplicity of mechanisms and affect additional molecular targets that may address symptoms and comorbidities of ASD. Here, we review the evidence for the use of metabolism-based therapies such as the KD in the treatment of ASD as well as emerging co-morbid models of epilepsy and autism. Future research directions aimed at validating such therapeutic approaches and identifying additional and novel mechanistic targets are also discussed.

## Autism spectrum disorder—complex etiology, limited therapies

Autism spectrum disorder (ASD) is characterized by persistent deficits in sociability and communication, as well as restricted and repetitive patterns of behavior and interests (DiCicco-Bloom et al., 2006; Llaneza et al., 2010; Lai et al., 2014). The term “spectrum” refers to the wide range of symptoms and levels of impairment that can occur in individuals with ASD. Beyond these core behavioral symptoms, ASD is increasingly shown to affect the gastrointestinal, immune, hepatic, and endocrine systems (Goines and Van de Water, 2010; Patterson, 2011; Hsiao, 2013; Frye et al., 2015; Mayer et al., 2015). Common co-morbidities include neurologic, psychiatric and physical conditions: neurologic comorbidities include epilepsy, sleep impairment, sensory abnormalities, and delays and/or deficits in motor function; psychiatric conditions such as depression, anxiety, irritability and attention deficit hyperactivity disorder; and physical health issues such as chronic gastrointestinal disturbance. The co-occurrence rate of one or more non-ASD developmental diagnoses is as high as 83% (Levy et al., 2010).

ASD occurs in all racial, ethnic, and socioeconomic groups and is highly prevalent. It affects tens of millions individuals worldwide and costs millions of US dollars on average to support an affected individual during his/her lifespan (Buescher et al., 2014). In the U.S., the incidence of ASD is 1 in 68 children (1 in 42 boys and 1 in 189 girls) based on data released by the Centers for Disease Control and Prevention (CDC) in 2014. The prevalence appears to be on the rise (a 10-fold increase in 40 years), and is explained only in part by improved diagnosis and awareness (Hansen et al., 2015). Developmental delay in ASD can be detected as early as 6 months of age, a critical time for the development of higher-order social, emotional, and communications functions (Courchesne et al., 2007); the importance of early intervention is recognized (Orinstein et al., 2014). However, on average, children are not diagnosed until after 4 years of age (CDC, 2014), even though patients can now be reliably diagnosed at 2 years of age (Lord et al., 2006; Kleinman et al., 2008).

Given that ASD has broad and heterogeneous manifestations, and has been associated with a plethora of possible etiological factors (both genetic and environmental), ASD remains a clinical and broad-spectrum diagnosis. In most cases, ASD is diagnosed without any defined etiology. A dearth of knowledge about underlying causes has limited the ability to develop and mobilize effective treatments, and currently only co-morbid manifestations of the disorder can be alleviated. The hope is that reducing co-morbidities such as epileptic seizures, psychiatric disturbances, hyperactivity, sleep problems and digestive issues (DiCicco-Bloom et al., 2006; Llaneza et al., 2010; Lai et al., 2014) will improve overall function and reduce the severity of ASD symptoms (Kohane et al., 2012; Frye and Rossignol, 2016).

Genetic susceptibility factors and environmental influences (and likely often both) contribute to ASD (Chaste and Leboyer, 2012; Sandin et al., 2014; Tordjman et al., 2014; Kim and Leventhal, 2015). Genome screening and sequencing has identified rare chromosomal abnormalities and copy number variations, as well as hundreds of rare gene mutations associated with autism (Devlin and Scherer, 2012; Huguet et al., 2013; Jeste and Geschwind, 2014; Baker and Jeste, 2015). A small number of these genetic changes appear highly penetrant and sufficient to cause autism. However, most genetic factors only increase the risk to varying degrees, and likely combine with additional influences such as advanced parental age at time of conception, adverse metabolic conditions and/or maternal illness during pregnancy, birth complications, and exposure to toxins and/or drugs during early brain development (Stromland et al., 1994; Durkin et al., 2008; Gardener et al., 2011; Krakowiak et al., 2012; Christensen et al., 2013). Not surprisingly, the molecular pathways implicated in ASD are also highly complex and diverse, and include synaptic dysfunction and plasticity of various neurotransmitter systems, transcriptional regulation and chromatin remodeling, protein translation and modification, neuroimmunological modulation, and mitochondrial function (Veenstra-Vanderweele et al., 2004; Bourgeron, 2015; De Rubeis and Buxbaum, 2015; Kopp et al., 2015; Loke et al., 2015; Mahfouz et al., 2015; Nelson and Valakh, 2015; Subramanian et al., 2015; de la Torre-Ubieta et al., 2016; Wen et al., 2016).

## Metabolism, mitochondria, and ASD

Given such extreme etiological diversity, it is reasonable to hypothesize that perturbation of a common nexus can precipitate the behavioral hallmarks of ASD (Geschwind, 2008; Berg and Geschwind, 2012). Identifying such a common factor would provide novel insights into the development of ASD. Further, targeting this pathway could lead to selective therapeutic approaches that might enhance efficacy and address core symptoms. One possibility is mitochondrial function, which is integral to many cellular pathways. In addition to its well-known role as the “powerhouse of the cell,” producing the bulk of the cellular energy, mitochondria are also critically involved in cellular metabolism, intracellular calcium signaling, generation of reactive oxygen species (ROS), and apoptosis (Suen et al., 2008; Murphy, 2009; Palmieri et al., 2010; Antico Arciuch et al., 2012; Rizzuto et al., 2012), as well as in the regulation of innate and adaptive immunity (Weinberg et al., 2015). For example, mitochondria carry out both cleavage and synthesis of glycine (Kikuchi et al., 2008), which is the ligand of glycine receptors. These receptors are chloride channels that mediate inhibitory neurotransmission in the adult nervous system. However, they are highly expressed in the embryonic brain and mediate excitatory neurotransmission, and are believed to promote cortical interneuron migration and generation of excitatory projection neurons (Pilorge et al., 2016). Interestingly, recent genetic and functional studies have identified a role of abnormal glycinergic signaling in ASD (Pilorge et al., 2016). Furthermore, mitochondria are known to be affected by many of the same endogenous and exogenous risk factors of ASD, such as toxins, drugs, immune activation, and metabolic disturbances (Frye and Rossignol, 2011). Thus, elucidating the role of mitochondrial dysfunction in ASD may help unify our understanding of this complex disorder.

Mitochondria play a particularly vital role in the central nervous system. The brain has very high energy demands, consuming approximately 20% of calories while accounting for only 2% of total body weight (Raichle and Gusnard, 2002), and demanding a great amount of adenosine triphosphate (ATP) to maintain ionic gradients essential for neurotransmission and plasticity (Harris et al., 2012). In addition, mitochondria are involved in the proliferation, differentiation and maturation of neural stem cells, formation of dendritic processes, developmental and synaptic plasticity, and cell survival and death (Li et al., 2004; Kann and Kovacs, 2007; Mattson et al., 2008; Kimura and Murakami, 2014; Xavier et al., 2016). Thus, it is not surprising that multiple lines of evidence in both human and animal models support a role for mitochondrial dysfunction in the etiology of ASD (Haas, 2010; Dhillon et al., 2011; Frye and Rossignol, 2011; Rossignol and Frye, 2012; Legido et al., 2013).

The prevalence of mitochondrial disease in the ASD population is estimated to be about 5.0%, 500 times higher than that found in the general population (≈0.01%). The prevalence of abnormal metabolic biomarkers is even higher, suggesting that as many as 30% of children with ASD may experience metabolic abnormalities: almost one-third of autistic children have documented elevations in plasma lactate and/or the lactate-to-pyruvate ratio, and the levels of many other mitochondrial biomarkers (pyruvate, carnitine, and ubiquinone) are significantly different between ASD and controls (Rossignol and Frye, 2012). In addition, several genes known to regulate mitochondrial function are clearly autism-risk genes. These include *SLC25A12* (Ramoz et al., 2004; Segurado et al., 2005; Silverman et al., 2008; Turunen et al., 2008; Kim et al., 2011), which encodes the predominant form of mitochondrial aspartate/glutamate carrier (however, also see Correia et al., 2006; Palmieri et al., 2010). These carriers participate in a wide range of mitochondrial functions, including control of respiration, calcium signaling and antioxidant defense, as well as glutamate-mediated excitotoxicity (Amoedo et al., 2016). Furthermore, *TMLHE*, (trimethyllysine hydroxylase epsilon), which encodes the first enzyme in carnitine biosynthesis, has also been associated with ASD (Celestino-Soper et al., 2012; Nava et al., 2012). It has been well established that carnitines are involved in mitochondrial transport of long-chain fatty acids and play an important role in maintaining normal mitochondrial function (Bremer, 1983). In addition, the gene encoding an inner mitochondrial membrane protease-like protein (*IMMP2L*) may help regulate susceptibility to ASD (Maestrini et al., 2010). It is important to note that metabolic and mitochondrial dysfunction may not exist in all patients with ASD, and biomarkers to identify this impairment would be advantageous in developing personalized treatment.

In parallel with clinical findings, many animal models of ASD also display mitochondrial dysfunction, including those based on susceptibility genes such as *MECP2, UBE3A, TSC*, and *FOXG1*. Mitochondrial dysfunction has also been observed in animal models of ASD induced by environmental risk factors such as maternal immune activation and exposure to propionic acid or valproic acid (VPA). Current evidence linking mitochondrial perturbations to ASD and the corresponding references are summarized in Tables 1, 2.

**Table 1:** **Studies showing linkage between ASD and mitochondrial dysfunction in ASD patients (only those reporting more than 25 subjects are included in this table)**.

**References**	**Cases**	**Evidence of mitochondrial dysfunction**
**GENETIC LINKAGE BETWEEN MITOCHONDRIA-RELATED GENES AND AUTISM**		
Celestino-Soper et al., 2012	909 or 130	Deficiency of the gene *TMLHE* (trimethyl-lysine hydroxylase epsilon), which encodes the first enzyme in carnitine biosynthesis, was more frequent in probands from male-male multiplex ASD families.
Glessner et al., 2009	859	Copy number variations in genes involved in the ubiquitin degradation were implicated in susceptibility for ASD.
Kent et al., 2006	129	The 3243A>G mitochondrial DNA mutation was concluded to be a rare cause of isolated Asperger syndrome.
Silverman et al., 2008; Kim et al., 2011	Multiple families	Polymorphism in *SLC25A12* gene, which encodes a mitochondrial aspartate/glutamate carrier, was found to be associated with restricted repetitive behaviors in autism.
Maestrini et al., 2010	127	A gene encoding an inner mitochondrial membrane protease-like protein (*IMMP2L)* was implicated in susceptibility for ASD.
Nava et al., 2012	501	Mutations in *TMLHE* were identified in patients with ASD and led to an increase in trimethyl-lysine, the precursor of carnitine biosynthesis, in the plasma.
Ramoz et al., 2004; Segurado et al., 2005; Turunen et al., 2008	Multiple families	Polymorphism in *SLC25A12* gene was found to be associated with autism.
**INDICATIONS OF IMPAIRED MITOCHONDRIAL FUNCTION IN THE BRAIN**		
Goh et al., 2014	75	Lactate doublets detected by brain magnetic resonance spectroscopic imaging were present at a higher rate in autistic patients.
Palmieri et al., 2010	Six or multiple families	Transport rates of mitochondrial aspartate/glutamate carrier (AGC) were higher in temporo-cortical gray matter. In addition, expression of AGC1, cytochrome c oxidase activity, and oxidized mitochondrial proteins were increased. However, variants of the AGC1-encoding *SLC25A12* gene were not correlated with AGC activation or autism phenotype.
Tang et al., 2013	45	Mitochondrial function and intracellular redox status were compromised in the pyramidal neurons of the temporal cortex.
**ABNORMAL LEVELS OF MITOCHONDRIA-RELATED METABOLITES IN BLOOD SAMPLES**		
Al-Mosalem et al., 2009	30	Increased plasma lactate levels and activity of creatine kinase.
Cohen et al., 1976	25	Increased serum creatine phosphokinase levels.
Correia et al., 2006	241	Increased plasma lactate levels and lactate/pyruvate ratio, but not associated with the variation at the *SLC25A12* gene.
Filipek et al., 2004	100	Reduced levels of carnitine and pyruvate, but increased levels of alanine and ammonia in serum.
Frye et al., 2013	213	Abnormal acyl-carnitine panels and glutathione metabolism in blood samples.
Kuwabara et al., 2013	25	Higher plasma levels of arginine and taurine, and lower levels of 5-oxoproline and lactic acid.
László et al., 1994	30	Increased serum lactate and pyruvate levels.
Moreno et al., 1992	60	Increased lactate and pyruvate levels.
Oliveira et al., 2005	69	20% of ASD patients showed significantly increased lactic acidemia, while 7% were classified with a definite mitochondrial respiratory chain disorder.
Poling et al., 2006	159	Increased blood aspartate aminotransferase and creatine kinase levels.
**ABNORMAL MITOCHONDRIAL FUNCTION AND DNA STRUCTURE IN PERIPHERAL CELLS OR CELL LINES**		
Boccuto et al., 2013	87	Decreased tryptophan metabolism in lymphoblastoid cell lines.
Chen et al., 2015	78	Mitochondrial DNA copy number in peripheral blood cells was elevated in children with ASD.
Rose et al., 2012	43	Primary immune cells in the blood had a more oxidized intracellular and extracellular microenvironment and a deficit in glutathione-mediated redox/antioxidant capacity.
Rose et al., 2014	25	Mitochondrial dysfunction observed in a subset of autism lymphoblastoid cell lines.
Wong et al., 2016	66	Mitochondrial DNA deletions and higher p53 gene copy ratios in peripheral blood monocytic cells were more common in children with autism and their fathers.

**Table 2:** **Studies showing association between ASD and mitochondrial dysfunction in animal models of ASD**.

**References**	**Evidence of mitochondrial dysfunction**
**LINKAGE BETWEEN MITOCHONDRIA-RELATED GENES AND AUTISTIC PHENOTYPE**	
Hullinger et al., 2016	Increased expression of AT-1/SLC33A1 caused an autistic-like phenotype in mice.
Inan et al., 2016	Progressive decline in oxidative phosphorylation led to circuit dysfunction, impaired sensory gating and social disability when the *cox10* gene was selectively deleted in parvalbumin neurons in mouse.
Sakurai et al., 2010	Loss of *SLC25A12* gene resulted in hypomyelination. Myelin deficits in slice cultures from knockout mice were reversed by administration of pyruvate.
Xie et al., 2016	Cell-autonomous insufficiencies in the activity of *TMLHE* reduced neural stem cell pools in the embryonic mouse brain.
Zhao et al., 2010	ASD-like features observed in neuronal glucose transporter isoform 3-deficient mice.
**ALTERATIONS IN MITOCHONDRIAL FUNCTION IN ANIMAL OR CELLULAR MODELS OF ASD BASED ON GENETIC FACTORS**	
De Filippis et al., 2015	The rate of hydrogen peroxide generation was increased and the function of complex ii impaired in the brain of *MeCP2-*308 heterozygous female mice.
Jin et al., 2015	*Mecp2*, whose mutations cause Rett syndrome, was observed to regulate mitochondrial bioenergetics through a glutamine transporter in microglia.
Kriaucionis et al., 2006	Mitochondrial abnormalities observed in *Mecp2*-null mouse, a model of Rett syndrome.
Nie et al., 2015	Mitochondrial uncoupling protein-2 was highly induced in *Tsc2*-deficient neurons, and also in a neuron-specific *Tsc1* conditional knock-out mouse model.
Norkett et al., 2016	DISC1 protein regulated mitochondrial dynamics in neurites of neurons.
Pancrazi et al., 2015	A fraction of the protein Foxg1, which is implicated in autism, was found to localize to mitochondria and coordinate cell differentiation and bioenergetics.
Santini et al., 2015	A mouse model of Angelman syndrome displayed elevated levels of mitochondria-derived reactive oxygen species in pyramidal neurons in hippocampal CA1 area, and administration of MitoQ, a mitochondria-specific antioxidant, to this model normalized synaptic plasticity and restored memory.
Su et al., 2011	Mitochondrial dysfunction observed in hippocampal neurons of the *UBE3A*-deficient mouse model of Angelman syndrome.
**MITOCHONDRIAL DYSFUNCTION IN ANIMAL MODELS OF ASD BASED ON ENVIRONMENTAL FACTORS**	
Bhandari and Kuhad, 2015	Propanoic acid exposure induced autism-like behavior in rats and activities of complex I and II were reduced.
Kumar and Sharma, 2016	Prenatal exposure to valproic acid decreased the activity of mitochondrial complex I, II, and IV in rats.
Macfabe, 2012	Mitochondrial dysfunction observed in a rat ASD model in which propionic acid, an enteric bacterial fermentation product, is infused intracerebroventricularly.
TREATMENT RELATED TO METABOLISM AND MITOCHONDRIAL FUNCTION IN ANIMAL MODELS OF ASD^*^	
Ciarlone et al., 2016	Ketone ester supplementation improved motor coordination, learning and memory, and synaptic plasticity in a mouse model of Angelman syndrome. The treatment also attenuated seizure activity and altered brain amino acid metabolism in this model.
Currais et al., 2016	Dietary glycemic index was found to modulate behavioral and biochemical phenotype of the BTBR mouse model of ASD.
Naviaux et al., 2013, 2014, 2015	Anti-purinergic therapy improved autism-like features in the maternal immune activation mouse model and the Fragile X mouse model.
Park et al., 2014	Dietary therapy with triheptanoin enhanced mitochondrial substrate use and improved metabolism and behaviors of *Mecp2*-null mouse model of Rett syndrome.
Sakurai et al., 2010	Loss of the *SLC25A12* gene resulted in hypomyelination. Myelin deficits in slice cultures from knockout mice are reversed by administration of pyruvate.
Santini et al., 2015	A mouse model of Angelman syndrome displayed elevated levels of mitochondria-derived reactive oxygen species in pyramidal neurons in CA1 hippocampus, and administration of MitoQ, a mitochondria-specific antioxidant, in this model normalized synaptic plasticity and restored memory.

* *Studies using the ketogenic diet are described in more detail in the main text*.

Common co-morbidities of ASD also suggest metabolic and mitochondrial dysfunction. One of the most significant co-morbidities is epilepsy, with a prevalence of 5–38% in children with ASD—much higher than the 1–2% prevalence in the general population (Frye, 2015). Seizures also occur in 35–60% of individuals with biochemically-confirmed mitochondrial disease (Rahman, 2012), suggesting there may be a common etio-pathology. Similarly, gastrointestinal dysfunction, a frequent comorbidity of ASD (Chaidez et al., 2014), is also common in mitochondrial disease (Frye et al., 2015).

Taken together, we believe that mitochondria act as a central nexus responding to and regulating many domains of cellular biology that have been implicated in ASD. Given the prevalence of metabolic/mitochondrial dysfunction in ASD, options for metabolic therapy should be explored. Below we review some of the emerging clinical and research evidence that metabolic therapy and improved mitochondrial function can ameliorate ASD symptoms and comorbidities.

## Metabolic therapy for ASD

A metabolic therapy in use for decades is the ketogenic diet (KD), a high-fat, low-carbohydrate diet—a remarkably effective non-pharmacological treatment for medically intractable epilepsy (Neal et al., 2008). Based on historical observations that either fasting or starvation rendered anti-seizure effects, the KD was designed to reproduce the biochemical changes seen in these physiological states (Masino and Rho, 2012). Recently, various dietary and metabolic therapies have been attempted in a wider variety of neurological diseases including ASD, Alzheimer's disease, Parkinson's disease, amyotrophic lateral sclerosis, sleep disorders, multiple sclerosis, brain trauma, stroke, pain, Huntington's disease and brain cancer (Ruskin et al., 2009; Stafstrom and Rho, 2012; Napoli et al., 2014). Although generally limited in scope, clinical studies thus far showed promising results in conditions such as Alzheimer's disease and ASD and are discussed in more detail below. In addition, research using animal models has pointed to a common mechanism of regulating energy metabolism to afford neuroprotective effects (Stafstrom and Rho, 2012).

Overall, recent clinical and laboratory evidence suggests that the KD may have positive effects in ASD. The complex pathophysiology of ASD and the diversity of mechanisms mobilized by dietary therapy combine to make identifying the key molecular mechanisms challenging, but a number of candidates are emerging. Two hallmark biochemical features after the KD treatment are increased ketone body production by the liver through fatty acid oxidation and reduced blood glucose levels (Stafstrom and Rho, 2012). More specific metabolic effects, such as increases in specific polyunsaturated fatty acids, might regulate neuronal membrane excitability (Voskuyl and Vreugdenhil, 2001), reduce inflammation (Cullingford, 2008; Jeong et al., 2011), or decrease the production of ROS by mitochondria (Kim do and Rho, 2008). Additionally, ketone bodies themselves have been shown to possess neuroprotective properties through improved bioenergetics - raising ATP levels and reducing ROS production through enhancement of NADH oxidation and inhibition of mitochondrial permeability transition (Kim do et al., 2007, 2015); related to this, a KD has also been shown to stimulate mitochondrial biogenesis (Bough et al., 2006; Ahola-Erkkila et al., 2010). In parallel, reduced glycolysis can suppress seizures, improve mitochondrial function, decrease oxidative stress, reduce activity of pro-apoptotic factors, and inhibit inflammatory mediators such as interleukins and tumor necrosis factor alpha (Garriga-Canut et al., 2006; Maalouf et al., 2009). The KD has also been proposed to increase adenosine (a product of extracellular ATP dephosphorylation); ATP and adenosine are purines with pleiotropic neuromodulatory and neuroprotective roles proposed to underlie in part the diet's clinical efficacy (Masino and Geiger, 2008; Masino et al., 2009, 2010). Separately, increased adenosine has been proposed to reduce symptoms and comorbidities of ASD (Masino et al., 2013). In addition, the KD has been reported to regulate energy-sensing pathways such as those involving the insulin-like growth factor and the mammalian target of rapamycin (McDaniel et al., 2011; Gano et al., 2014). Epigenetic regulation is a new but potentially important mechanism as well (Boison, 2016).

Given the effects of the KD and its substrates (e.g., ketone bodies, fatty acids) on cognitive and behavioral functioning, it is reasonable to speculate that this diet would induce changes in synaptic morphology and function. Studies have shown that the KD can modulate excitability through actions on potassium ion channels (Tigerholm et al., 2012; Lutas and Yellen, 2013) and glutamatergic synaptic transmission (Xu et al., 2006; Juge et al., 2010; Lutas and Yellen, 2013; Chang et al., 2016), as well as possible regulation of GABA production (Yudkoff et al., 2007). In addition, the KD or its metabolic mediators can induce changes in synaptic vesicular cycling (Hrynevich et al., 2016), hippocampal mossy fiber sprouting (Muller-Schwarze et al., 1999), and both age- and region-dependent changes in synaptic morphology (Balietti et al., 2008, 2009).

Collectively, evidence thus far indicates that the KD affords broad neuroprotective effects, and hence, it is reasonable to hypothesize that this diet could prove to be beneficial for individuals with ASD.

## Metabolic therapy and ASD—clinical evidence to date

To date, there have been limited clinical trials involving treatment of ASD patients with metabolic therapy using variants of a KD. The first report was a pilot prospective study in autistic children aged between 4 and 10 years carried out by Evangeliou and colleagues; they applied an intermittent modified medium-chain triglyceride (MCT) diet (Evangeliou et al., 2003). Most of the 18 patients who adhered to the diet improved based on the Childhood Autism Rating Scale (CARS) and several additional clinical parameters. Significant (i.e., >12 units of decrease in CARS) and average (>8–12 units of decrease in CARS) improvement was recorded in two and eight patients, respectively, while minor (2–8 units of decrease in CARS) improvement was reported in the remaining eight patients. More recently, Spilioti and colleagues reported the effects of KD treatment in a group of Greek children with ASD aged between 3.5 and 6 years (Spilioti et al., 2013). Of the 6 patients who implemented the diet successfully, significant and average improvement was recorded in one and two patients, respectively, and minor improvement was reported in the remaining three patients.

The diet is also effective in reducing common comorbidities of ASD such as seizures, not surprisingly, but also improved cognition and behavior. A pilot retrospective study analyzed outcomes in children prescribed the KD to treat epileptic seizures; among these children, some also had autistic symptoms and abnormal behaviors. Children assigned in the KD group were currently on the diet and had been for at least 6 months; children assigned in the non-KD group stopped the diet at least 2 months prior. Fewer abnormal behaviors and significant behavioral improvement were found in the KD group, and behavioral improvement was not correlated with seizure control (Masino et al., 2011). More recently a randomized control trial showed improved cognition, mood and behavior—particularly reduced anxiety—in children prescribed the KD for refractory epilepsy. These behavioral benefits were also unrelated to seizure control (IJff et al., 2016). In a remarkable case study, Herbert and Buckley reported on a 12-year-old child with comorbid autism and epilepsy treated with a gluten- and casein-free KD (fats composed mostly of MCTs) (Herbert and Buckley, 2013). In addition to a significant reduction in seizures, the diet resolved morbid obesity and improved cognitive and behavioral function. Over the course of several years following initial diagnosis, the child's CARS score decreased from 49 to 17, representing a change from severe autism to a non-autistic state, and her intelligence quotient increased by 70 points.

In summary, clinical evidence to date remains limited, but results from the aforementioned studies show promise that metabolic therapy with several different versions of a KD can improve symptoms of ASD and can also improve cognition and behavior—the latter benefits that can facilitate optimal outcomes in ASD. In patients with diagnosed ASD, greater than 50% of autism patients who received this metabolic therapy showed moderate-to-significant clinical improvement, while the remainder displayed minor improvement. At present, more larger-scale clinical studies are required. Meanwhile, as mentioned earlier, metabolic and mitochondrial dysfunction may represent only a subgroup of the ASD population. Thus, it would be important to determine the relation between the effects of the KD and metabolic/genetic profile of ASD patients.

## Metabolic therapy and ASD—evidence from animal models

Due to the complexity of ASD, investigators have developed and employed numerous animal models. Some have clear metabolic underpinnings, underscoring the link between metabolic dysfunction and symptoms of autism. Metabolic therapy with a KD and/or a restricted diet has already been examined in several models. In agreement with the aforementioned clinical studies, reports in animal models have been positive. The ASD models tested with metabolic therapy discussed here include genetic disorders that mirror clinical conditions, induced ASD that models environmental conditions found to increase ASD risk in humans, and behavioral ASD models with unknown etiologies that recapitulate all or some of the core symptoms, and may or may not have comorbid seizures.

As one genetic example, succinic semialdehyde dehydrogenase (SSADH) deficiency is a rare autosomal recessive condition that results in mild-to-moderate mental retardation, disproportionate language dysfunction, seizures, hypotonia, hyporeflexia, hallucinations, and autistic behaviors (Pearl et al., 2003). In an animal model of SSADH deficiency, the SSADH knockout mouse, Nylen and colleagues found that KD treatment normalized electroencephalogram (EEG) activity and restored miniature inhibitory post-synaptic currents recorded in CA1 pyramidal cells using hippocampal slices. In contrast, there were no significant differences between the groups in terms of miniature excitatory post-synaptic currents. Behaviorally, KD-treated mutant animals experienced significantly fewer seizures compared to mutant animals fed the control diet (Nylen et al., 2008).

Metabolic therapy with dietary restriction (either a standard diet or KD) was tested in another clinically relevant genetic model of Rett syndrome. Rett syndrome is a neurodevelopmental disorder characterized by normal early maturation, followed by a slowing of development, impairment of motor functions, seizure susceptibility, and intellectual disability. In most cases, Rett syndrome is caused by mutations in the methyl-CpG-binding protein 2 (*MECP2*) gene (Amir et al., 1999). Children with Rett syndrome often exhibit autistic-like behaviors in the early stages of the disease (Percy, 2011). Mantis and colleagues found that *Mecp2* mutant mice performed significantly worse in assays of motor function and anxiety compared to wild-type control animals, and restriction of either standard diet or the KD improved motor behavior and reduced anxiety in these mutant animals (Mantis et al., 2009). There is also limited clinical evidence for anti-seizure efficacy and improved behavior after KD treatment in Rett syndrome (Liebhaber et al., 2003).

Most cases of ASD have unknown genetic underpinnings (Gaugler et al., 2014), and models of unidentified etiology have been characterized with behavioral tests assessing autistic symptomatology. The BTBR T+tf/J (BTBR) inbred mouse strain is one of the most clinically relevant animal models of autism; it was identified in an extensive effort to characterize ASD-like behaviors in ten inbred mouse strains (Moy et al., 2007) and displays all the core behavioral features that define the disorder (Moy et al., 2007; McFarlane et al., 2008; Meyza et al., 2013; Ruskin et al., 2013; Smith et al., 2014; Ellegood and Crawley, 2015). BTBR mice display deficits in social interaction and communication assays and exhibit repetitive and stereotyped behaviors. During the relatively short time since its discovery as an ASD model, the BTBR strain has been increasingly used to study the etiology and to uncover potential interventions for ASD (Moy et al., 2007; McFarlane et al., 2008; Llaneza et al., 2010; Ruskin et al., 2013; Cheng et al., 2016; Mychasiuk and Rho, 2016; Newell et al., 2016). Ruskin and colleagues reported that the BTBR mice showed decreased sociability in the three-chamber test, decreased self-directed repetitive behavior, and improved social communication in a food preference assay after being fed a KD (Ruskin et al., 2013). In addition, the authors showed that the behavioral improvements were probably not related to any anti-seizure effect of the diet, because no spontaneous seizures or abnormal EEG features were observed in the BTBR animals.

Interestingly, a recent study showed that gut microbiota composition of cecal and fecal samples was significantly altered in BTBR mice compared to B6 animals (Newell et al., 2016). In addition, a KD decreased total host bacterial abundance in both sample types, and in the BTBR animals counteracted a low Firmicutes to Bacteroidetes ratio, which is commonly observed in patients with ASD (Finegold et al., 2010; De Angelis et al., 2013). Related to these findings, it has been shown that dietary glycemic index, which is a measure of how much the carbohydrate in a food item affects blood glucose level, modulates behavioral and biochemical phenotype in the BTBR mice (Currais et al., 2016). These data support the idea that in the context of genetic predisposition to ASD, diet could potentially alter the expression of the disorders.

More recently, Ruskin et al. also showed KD-induced behavioral improvements in the EL mouse, a model of comorbid ASD-associated behaviors and progressive spontaneous epilepsy. Mice (of both sexes) were fed a KD for 3 weeks after weaning and prior to the age of onset of the seizure phenotype. Sociability improved and repetititve behaviors decreased; intriguingly, these effects were more pronounced in females. Also, some behavioral benefits were observed in females even when a more liberal dietary formulation was applied (Ruskin et al., 2016).

As mentioned above, environmental factors also contribute to the risk of developing ASD. In this regard, exposure to exogenous chemicals is best exemplified by VPA use during pregnancy. VPA is a pharmacological anticonvulsant used in humans primarily for the treatment of epilepsy and migraine, and epidemiological studies have shown that use of VPA during pregnancy is associated with an increased risk of ASD in the offspring (Bromley et al., 2013; Christensen et al., 2013). The VPA exposure model is one of the most frequently studied models of autism (Chomiak et al., 2013; Roullet et al., 2013) since it exhibits many similar structural and behavioral features seen in ASD individuals. Ahn and colleagues found that KD treatment recovered part of the play behavior of juvenile rats exposed to VPA prenatally (Ahn et al., 2014). Interestingly, the authors also found that prenatal exposure to VPA altered mitochondrial respiration, and the KD was able to partially restore this. A recent study in VPA-treated mice also found improved social behavior (Castro et al., 2016).

Taken together, there is increasing evidence for the beneficial effects of the KD in different animal models of ASD. However, clear evidence for converging mechanistic links remains hypothetical, and few fundamental mechanistic studies have been conducted to date in either animal models or human ASD tissues. Thus, there is a need for further studies utilizing diverse animal models and incorporating comprehensive behavioral assays to elucidate common molecular pathways in ASD and to validate the positive effects of the KD observed thus far in animal models. Equally important, studies aimed at identifying the mechanisms relevant to such models of ASD are required to optimize treatment, discover novel therapeutic targets, and ultimately provide key insights to the neurobiology of ASD. Borrowing from the rich literature on the KD in epilepsy, shifts in energy metabolism, the direct actions of the ketone bodies on the mitochondria, neuromodulatory functions of ATP and adenosine, regulation of excitation/inhibition balance, and epigenetic effects of the diet are among the promising candidate mechanisms.

## Future directions: metabolic therapy and ASD

At present, there is strong evidence that mitochondrial and metabolic dysfunction may underlie the complex pathophysiology of ASD. Precise mechanisms remain elusive and many questions remain unanswered: Is mitochondrial dysfunction a cause or a consequence of ASD? In which specific organs and cell types is it most relevant? Can addressing mitochondrial and metabolic disturbances directly help at least a subgroup of patients with ASD for more targeted treatments that will ameliorate the diverse symptom complex? Thus far, the KD is a proven metabolic therapy for medically intractable epilepsy, but there are only limited data for its use in ASD and a rudimentary understanding of how the KD may exert positive behavioral effects. The optimum formulation of the KD needs to be established and may be different for ASD compared to epilepsy. Proper efforts to address these fundamental questions and to identify molecular mechanisms and biomarkers will require the collective and collaborative efforts of many, including basic, translational and clinical researchers, as well as investigators with diverse expertise in multi-organ dysfunction, metabolism, and genetic and environmental risk factors. The ultimate reward could be a major breakthrough in understanding its causes and developing much-needed broadly effective therapies for ASD—and in particular, treatments that address core symptoms.

## Author contributions

All authors have drafted and revised the manuscript together and approved it for publication.

### Conflict of interest statement

The authors declare that the research was conducted in the absence of any commercial or financial relationships that could be construed as a potential conflict of interest.
